# Subluminal group velocity and dispersion of Laguerre Gauss beams in free space

**DOI:** 10.1038/srep26842

**Published:** 2016-05-27

**Authors:** Nestor D. Bareza, Nathaniel Hermosa

**Affiliations:** 1 National Institute of Physics, University of the Philippines Diliman, Quezon City, Diliman 1101, Philippines

## Abstract

That the speed of light in free space *c* is constant has been a pillar of modern physics since the derivation of Maxwell and in Einstein’s postulate in special relativity. This has been a basic assumption in light’s various applications. However, a physical beam of light has a finite extent such that even in free space it is by nature dispersive. The field confinement changes its wavevector, hence, altering the light’s group velocity *v*_*g*_. Here, we report the subluminal *v*_*g*_ and consequently the dispersion in free space of Laguerre-Gauss (*LG*) beam, a beam known to carry orbital angular momentum. The *v*_*g*_ of *LG* beam, calculated in the paraxial regime, is observed to be inversely proportional to the beam’s divergence *θ*_0_, the orbital order ℓ and the radial order *p*. *LG* beams of higher orders travel relatively slower than that of lower orders. As a consequence, *LG* beams of different orders separate in the temporal domain along propagation. This is an added effect to the dispersion due to field confinement. Our results are useful for treating information embedded in *LG* beams from astronomical sources and/or data transmission in free space.

Recently, Giovannini *et al*. showed thru experiments, backed by calculations, that spatially structured light indeed travels slower than *c* within a certain path distance^1^. That is, there is a decrease of group velocity *v*_*g*_ for structured light. Although the phenomenon can be explained classically, they used a Hong-Ou-Mandel interferometer to measure the lag of a laterally structured photon compared to a photon with little lateral structure. In their experiment, the slowing of light is due to dispersion in free space. They performed their experiment with a Bessel beam and a Gaussian beam. Alfano and Nolan remarked that by considering dispersion relation, Bessel beam can be very slow near a critical frequency which can be used as optical buffer in free space^2^. Slowing light due to its structure is different from slowing light with materials.

Laguerre-Gauss (*LG*) beam is an interesting structured light since it carries orbital angular momentum (OAM). *LG* beam can have orders of orbital or winding order 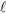 and radial order *p*. The scalar field of *LG* beam is expressed mathematically in standard cylindrical coordinates (*r*, *φ*, *z*) as follows,


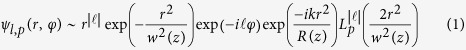


where 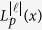 is the generalized Laguerre polynomial with 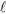 as integers and *p* ≥ 0, 
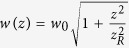
 is the beam waist, 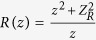 is the radius of curvature, and 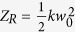 is the Rayleigh length^3^. This beam spreads along propagation as illustrated in Fig. 1a. In the far-field, the beam divergence of an 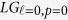 is represented by the opening angle *θ*_0_, which can be expressed in terms of minimum beam waist *w*_0_ and magnitude of the wavevector *k*_*0*_ as,


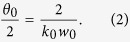


In the *LG* expression in (1), the phase factor of the form 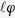 means 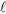 is the number of 2*π* windings around the azimuthal angle *φ*. First asserted by Allen *et al*., these beams 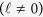 have Poynting vectors that spiral along the direction of propagation^4^. The helical wavefront for a beam with 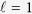 is illustrated in Fig. 1b. Negative 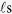 will yield the same wavefronts but of opposite helicities. The realization that *LG* beam carries OAM has led to a myriad of applications from optical tweezing and micromanipulation^5^,^6^,^7^, to free space information^8^, to tranverse Doppler effect^9^, and in astrophysics^10^.

Although the radial order *p* of *LG* mode is very rarely discussed, it is mostly directly used in applications. One fundamental role of *p* is its enhancement of the angular beam shifts in reflection of higher order *LG* beams^11^. Moreover, higher orders of *LG* modes are also found to reduce the Brownian thermal noise in laser interferometry that could be useful in future gravitational wave detectors^12^. In optical trapping, if an optical vortex due to 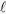 confines atom for precision measurements, the multi-ring dislocations due to *p* can be used as toroidal trap in observing persistent flow of Bose-Einstein condensates^13^,^14^.

In the paraxial regime, *LG* beams form a complete basis set such that it can be used as a tool in quantum information processes^15^,^16^,^17^. Both 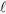 and *p* are realized as additional degrees of freedom in encrypting information in photons^18^,^19^. Hence, both the orbital and radial order can be used in encoding information aside from the polarization of the light. As an application, free-space multiplexing is possible as photons are treated with higher quantum dimensional states. Consequently, higher information density can be achieved even using the same number of photons.

In this manuscript we ask: *What is the effect of the orbital order* 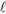 *and the radial order p of LG beam on its group velocity?* The consequences are extensive. The most important of which is the different time of arrival of information even in free space propagation. This is similar to the modal dispersion in fiber, a serious limitation in optical fiber communication^20^. The promised massive information when using *LG* beams will have an issue. Information embedded in these beams will not arrive at the same time and some corrections are then necessary.

In this paper, we report our calculation on the dispersion and reduction of *v*_*g*_′s in *LG* beams. The analytical expression is exact and our expression reduces to the result of Giovannini *et al*. for Gaussian beam when 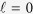 and *p* = 0.

## Results and Discussions

The *v*_*g*_ can be derived by considering geometry in the ray-optic model. The path of light follows the direction of Poynting vector which points toward the direction of the wavevector. A field confinement produces spatially structured light, which alters the wavevector to include non-axial components. The transverse components cause the delay in the *v*_*g*_ of light. Confined light therefore, would have its *v*_*g*_ that is not equal to *c*.

Suppose light travels along *z* in standard cylindrical coordinates (*r*, *φ*, *z*). A plane wave has a wavevector component that is purely along *z* thus, this light is expected to travel at *c*. For Gaussian and Bessel beams, the wavevectors comprise of both longitudinal *z* and radial *r* components. The radial component will cause an added path length in the propagation of these beams. It will generate a time delay in the speed of light. For beams with OAM, the wavevectors constitute the whole basis components. The delay then for an OAM-carrying beam is due to the added path length that originated from both radial and azimuthal wavevector components.

The *v*_*g*_ calculation in the paraxial regime of *LG* beam is detailed in the *Methods* section. The *v*_*g*_ is found to be inversely proportional to the orbital order 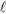, the radial order *p*, and the beam’s divergence *θ*_0_, as


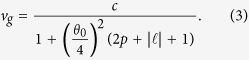


This expression shows that the delay of *LG* beam is related to its order, 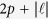. When the order is zero, the beam reduces to a Gaussian mode 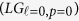. The *v*_*g*_ for 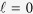, *p* = 0 is consistent with the reported delay in Gaussian beams^1^. The subluminal *v*_*g*_ of Gaussian modes varies for different *w*_0_ values, and that *v*_*g*_ is even further reduced for relatively smaller *w*_0_. This holds true since, for a certain *λ*_0_, relatively lower *w*_0_ yields larger far-field beam divergence. As the beam propagates for such case, the field confinement in the transverse structure is amplified.

For a fixed *θ*_0_, the expression results with discrete *v*_*g*_ values, since 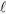 and *p* take the values of integers and natural numbers, respectively. This fact is helpful for precise detection in communications using *LG* beams, as one has prior knowledge of the beams’ arrival based on discrete *v*_*g*_’s.

**Figure 2:**
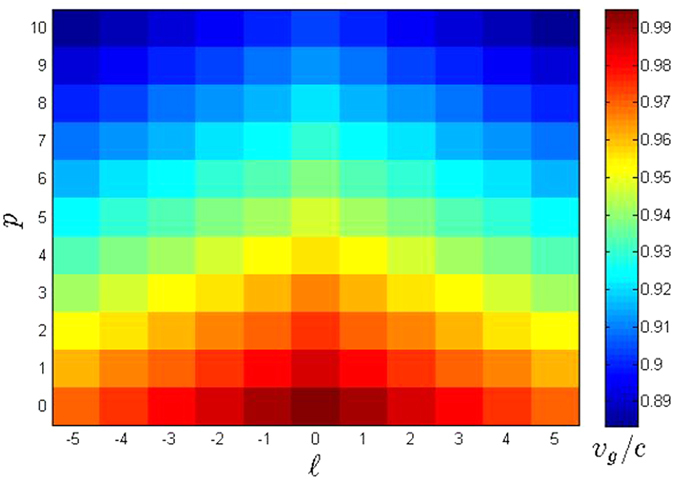
Colormap of *v*_*g*_/*c* values as function of 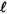 and *p* with central wavelength *λ*_0_ = 632.8 *nm* and minimum beam waist *w*_0_ = 2.0 *μm*. Each pixel of a specific color corresponds to *v*_*g*_/*c* value (colored scale bar). Warm colored pixels have relatively higher *v*_*g*_/*c* values compared to cool colored pixels.

As a representation of Equation (3), a colormap of *v*_*g*_/*c* values for 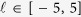 and *p* ∈ [0, 10], is shown in Fig. 2. We generated this plot with a beam of a central wavelength *λ*_0_ = 632.8 *nm* and a minimum beam waist *w*_0_ = 2.0 *μm*. All values fall below unity implying subluminal speed of *LG* beams for any 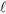 and *p* values. The case 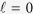 and *p* = 0, located at the center of lowest row, corresponds to *v*_*g*_/*c* of a Gaussian beam. This beam obtained the largest *v*_*g*_/*c* value or the least reduced *v*_*g*_. This is expected since a Gaussian beam with no radial and orbital order is the least structured beam compared to higher modes of *LG* beams. A Gaussian beam yields the least magnitude of transverse component in the altered wavevector, hence it intuitively results with *v*_*g*_ closest to *c*.

The *v*_*g*_/*c* becomes lower as one goes farther from 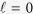 and *p* = 0, seen by the change in the color in Fig. 2. Different orders 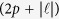 of *LG* beams disperse along propagation. The free-space dispersion based on Equation (3) can be expressed as the effective group index of refraction *n*_*g*_, given by, 
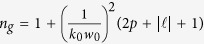
. For any *w*_0_ values, *n*_*g*_ is linearly related to 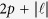. Thus, *LG* beams of different orders that are initially propagated simultaneously will have different time delays after travelling the same path distance. This makes *LG* beams separate in the temporal domain. This contributes to the dispersion due to field confinement. A beam with higher order will have greater added path length *δz*, evident when relating Equation (13) to Equation (10) (*see derivation in Methods section*).

The free-space dispersion of *LG* beams consequently demands corrections in their applications such as in data transmission/communication, in multiplexing, in interaction with nonlinear materials and in OAM spectrum detection^21^,^22^,^23^,^24^,^25^,^26^. The dispersion can also be substantial in quantum information processes for encryption and decryption of higher quantum dimensional states, such as 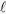 and *p* values, in photons.

Setting *p* = 0 in Equation (3), the role of different values of OAM alone can be seen. Padgett *et al*. demonstrate that for a given beam size, the far-field opening angle increases with increasing OAM^27^. Larger apertures are required when receiving beams with relatively higher OAM. The 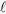-dependence of *v*_*g*_ for *LG* beams that we report may be incorporated to such receiving optical system. A time-controllable receiving aperture size can be programmed according to computed delays prior to the arrival of beams. As opposed to the beam divergence relation presented in Equation (2) due to skewness of Poynting vector with respect to optical axis, they also considered the contribution of normal diffractive spreading by the standard deviation of the spatial distribution. They derived the far-field beam divergence 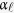 to be dependent on 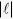 whose relation is given by, 
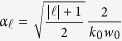
. Reformulating Equation (11), the *v*_*g*_ expression for OAM-carrying beams (*p* = 0) according to this beam divergence definition, we get a more compact form:


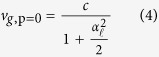


For light with OAM 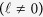 and *p* = 0, we can think that the added path length due to beam divergence increases by a factor of 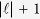. This factor is consistent with the conservation of total linear momentum in the system. In the work of Giovannini *et al*.^1^, the added path length comes from the radial component of the Poynting vector with respect to the optical axis. In Equation (13) (*see Methods section*), we show that even a Poynting vector with angular component due to 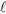 with respect to the optical axis can also contribute to the path.

**Figure 3:**
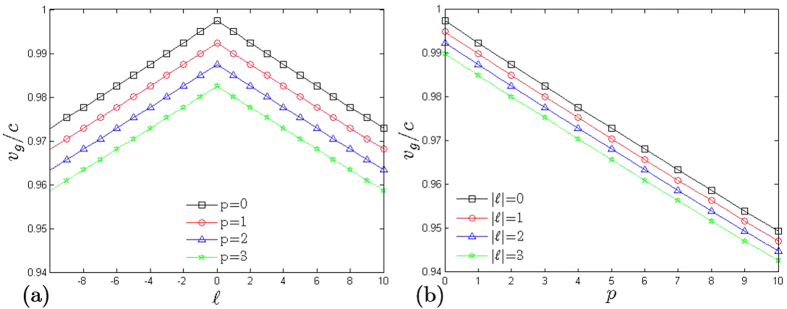
Plots of (**a**) *v*_*g*_/*c* versus 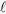 for different *p* values and (**b**) *v*_*g*_/*c* versus *p* for different 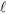 values.

Figure 3a shows the plots of *v*_*g*_/*c* versus 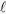 for different *p* values. The symmetry of trends between 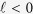 and 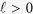 with respect to 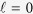 shows that the dispersion of OAM-carrying beams yield the same value of *v*_*g*_ regardless of the helicity or polarity of 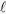. In Fig. 2, the color distributions between left and right regions mirror each other with respect to the central column, owing to the 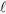 factor in Equation (3). The plot is shifted downwards for relatively higher radial order (*p* > 0). The *v*_*g*_ is reduced by an added 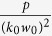 factor in the denominator of Equation (4).

Similarly, *v*_*g*_/*c* is plotted against *p* for different 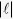 values in Fig. 3b. The drop in *v*_*g*_/*c* values in these plots is steeper compared to plots of *v*_*g*_ versus 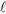. This is due to the 2 factor in *p* in Equation (3). Beams of different radial orders disperse faster than beams of different OAM. The plot of *v*_*g*_/*c* versus *p* shifts downward as the beam is endowed with higher orbital order.

Different modes can have the same *v*_*g*_ as seen in Fig. 3. These modes have the same beam order but of different combinations of mode indices. We call these modes with the same *v*_*g*_ as degenerate modes. There will be more degenerate modes for lower *v*_*g*_. This can be seen if we include more plots for higher values of *p* (>3) in Fig. 3a. The same can be observed in Fig. 3b by including plots with higher 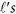, except that twice the modes must be accounted for 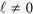 to consider the opposite helicities. Relatively higher beam order yields more degenerate modes.

The number of degenerate modes, denoted by 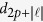, in the dispersion of *LG* beam with 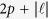 order is given by,


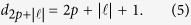


Only the Gaussian beam is non-degenerate, which uniquely is the fastest relative to other *LG* modes. The number of degenerate modes is just one plus the order of the beam. Some combinations of mode indices that yield the same *v*_*g*_ are presented in Table 1. In detection, the order of *LG* beam can be determined by performing cross correlation function even with intensity that resulted from partially coherent source^28^. There are several ways to discriminate the explicit combination of mode indices in degeneracy of the beam order. One example is to first quantify *p* by employing double correlation function on the captured intensity profile^29^. Then, the magnitude and polarity of 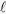 can be characterized by measuring OAM based on Fraunhofer diffraction pattern that is formed by passing light through shaped apertures^30^,^31^.

In conclusion, we have derived the group velocity *v*_*g*_ of *LG* beam that is inversely proportional to the orbital order 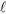, the radial order *p* and the far-field beam divergence *θ*_0_. This result shows that *LG* beams are both subluminal and dispersive even in free space. Discrete 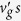 are obtained for an arbitrary *θ*_0_. The dispersion of *LG* beams has degenerate modes for certain discrete *v*_*g*_; The number of degenerate modes is just one plus the *LG* beam’s order 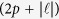. We also highlight that light travels in the direction of the Poynting vector, therefore both radial and angular components will contribute to the added path length. This report would have far-reaching consequences on the OAM beam’s applications.

## Methods

The transverse wavevector of a light beam alters both the phase velocity *v*_*p*_ and group velocity *v*_*g*_. We are only concern with *v*_*g*_ calculation since this parameter corresponds to the actual speed of light as it travels through space, whereas *v*_*p*_ indicates the field signal variation^32^.

For a given path length Δ*z* between two different points such as *z*_1_ and *z*_2_, a structured light travels at a time Δ*t* that includes an added path length *δz* due to the transverse components of the wavevector. They are related by Δ*t* = (Δ*z* + *δz*)/*c*. In the ray-optic model, the *v*_*g*_ can be obtained by calculating *δz* and is mathematically formulated as follows,


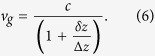


The path of light can be represented as a diverted ray with a certain angle from the beam axis. The amount of *δz* is the difference between length of diverted ray within the actual path and Δ*z*. This can be expressed as


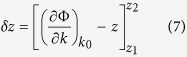


where Φ is the phase profile of the scalar field. As an example, Φ = *kz* − *ωt* for plane wave thus *δz* is zero, as expected. However for *LG* beam, the beam waist varies significantly at distances near the Rayleigh length. This manifests variation of *v*_*g*_ as it propagates in the near field. We consider the paraxial regime in order to simplify further *δz*, so that we can derive an expression of *z*-independent *v*_*g*_ for any arbitrary field. This then translates spatial dependence of *v*_*g*_ into wavevector. The derivation by Giovannini *et al*.^1^ considers Φ as complex argument of the scalar field function,


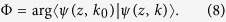


The paraxial wave equation is then written in terms of quantum mechanical operator with evolution of wavefunction from *z*_1_ to *z*_2_:


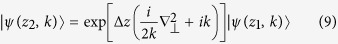


where 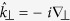 is the operator representing the transverse wave vector. By taking an inner product of *ψ*(*z*_2_, *k*) in Equation (9) and substituting the result to Equation (7), we obtain the relation:


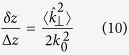


so that Equation (6) becomes,


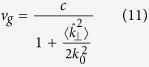


where,


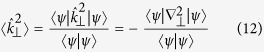


such that 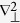 operator is the transverse Laplacian.

Now, for an *LG* beam, we substitute Equation (1) to Equation (12) in order to have


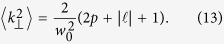


And the group velocity for such beam is given by,


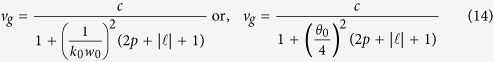


where 1/*k*_0_*w*_0_ is replaced by the opening angle of the beam *θ*_0_/4 for a more intuitive picture. When 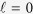 and *p* = 0,


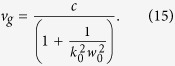


Equation (15) is consistent with the calculation for Gaussian beam^1^.

## Additional Information

**How to cite this article**: Bareza, N. D. and Hermosa, N. Subluminal group velocity and dispersion of Laguerre Gauss beams in free space. *Sci. Rep*. **6**, 26842; doi: 10.1038/srep26842 (2016).

## Figures and Tables

**Figure 1:**
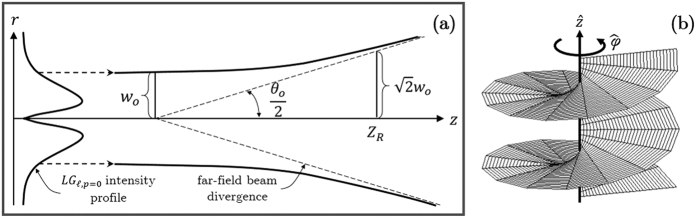
(**a**) *LG* beam spreading through propagation and (**b**) *LG* wavefront for 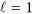 and *p* = 0.

**Table 1:** Some combinations of mode indices yielding 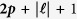 degenerate *v*_*g*_ values of *LG* beam in 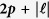 order.

Order 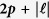	Degeneracy counts 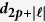	Mode indices 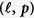
1	2	(1, 0), (−1, 0)
2	3	(2, 0), (−2, 0), (0, 1)
3	4	(3, 0), (−3, 0), (−1, 1), (1, 1)
4	5	(4, 0), (−4, 0), (2, 1), (−2, 1), (0, 2)
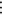	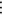	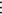

## References

[b1] Giovannini D. . Spatially structured photons that travel in free space slower than the speed of light. Science 347, 857–860 (2015). 25612608 10.1126/science.aaa3035

[b2] Alfano R. R. & Nolan D. A. Slowing of bessel light beam group velocity. Opt. Commun. 361, 25–27 (2016).

[b3] Mazilu M. & Dholakia K. Twisted photons: applications of light with orbital angular momentum (eds Torres J. P., Torner L.) Ch. 4, 37–62 (John Wiley & Sons, 2011).

[b4] Allen L., Beijersbergen M. W., Spreeuw R. & Woerdman J. Orbital angular momentum of light and the transformation of laguerre-gaussian laser modes. Phys. Rev. A 45, 8185 (1992). 9906912 10.1103/physreva.45.8185

[b5] Curtis J. E., Koss B. A. & Grier D. G. Dynamic holographic optical tweezers. Opt. Commun. 207, 169–175 (2002).

[b6] Galaja P. . Twisted photons: applications of light with orbital angular momentum (eds Torres J. P., Torner L.) Ch. 7, 117–139 (John Wiley & Sons, 2011).

[b7] Simpson N., Dholakia K., Allen L. & Padgett M. Mechanical equivalence of spin and orbital angular momentum of light: an optical spanner. Opt. Lett. 22, 52–54 (1997). 18183100 10.1364/ol.22.000052

[b8] Gibson G. . Free-space information transfer using light beams carrying orbital angular momentum. Opt. Express 12, 5448–5456 (2004). 19484105 10.1364/opex.12.005448

[b9] Rosales-Guzmán C., Hermosa N., Belmonte A. & Torres J. P. Experimental detection of transverse particle movement with structured light. Sci. Rep. 3 (2013). 10.1038/srep02815 PMC3788373 24085150

[b10] Berkhout G. C. & Beijersbergen M. W. Method for probing the orbital angular momentum of optical vortices in electromagnetic waves from astronomical objects. Phys. Rev. Lett. 101, 100801 (2008). 18851201 10.1103/PhysRevLett.101.100801

[b11] Hermosa N., Aiello A. & Woerdman J. Radial mode dependence of optical beam shifts. Opt. Lett. 37, 1044–1046 (2012). 22446218 10.1364/OL.37.001044

[b12] Chelkowski S., Hild S. & Freise A. Prospects of higher-order laguerre-gauss modes in future gravitational wave detectors. Phys. Rev. D 79, 122002 (2009).

[b13] Kennedy S. A., Szabo M. J., Teslow H., Porterfield J. Z. & Abraham E. Creation of laguerre-gaussian laser modes using diffractive optics. Phys. Rev. A 66, 043801 (2002).

[b14] Ryu C. . Observation of persistent flow of a bose-einstein condensate in a toroidal trap. Phys. Rev. Lett. 99, 260401 (2007). 18233561 10.1103/PhysRevLett.99.260401

[b15] Mair A., Vaziri A., Weihs G. & Zeilinger A. Entanglement of the orbital angular momentum states of photons. Nature 412, 313–316 (2001). 11460157 10.1038/35085529

[b16] D’Ambrosio V., Nagali E., Marrucci L. & Sciarrino F. Orbital angular momentum for quantum information processing. In SPIE Photonics Europe, 84400F–84400F (International Society for Optics and Photonics, 2012).

[b17] Molina-Terriza G., Torres J. P. & Torner L. Twisted photons. Nat. Phys. 3, 305–310 (2007).

[b18] Karimi E. . Exploring the quantum nature of the radial degree of freedom of a photon via hong-ou-mandel interference. Phys. Rev. A 89, 013829 (2014).

[b19] Djordjevic I. B. Deep-space and near-earth optical communications by coded orbital angular momentum (oam) modulation. Opt. Express 19, 14277–14289 (2011). 21934792 10.1364/OE.19.014277

[b20] Verdeyen J. T. Laser electronics (Englewood Cliffs, NJ, Prentice Hall, 1989).

[b21] Wang J. . Terabit free-space data transmission employing orbital angular momentum multiplexing. Nat. Photonics 6, 488–496 (2012).

[b22] Karimi E., Marrucci L., de Lisio C. & Santamato E. Time-division multiplexing of the orbital angular momentum of light. Opt. Lett. 37, 127–129 (2012). 22854442 10.1364/OL.37.000127

[b23] Tamburini F. . Encoding many channels on the same frequency through radio vorticity: first experimental test. New J. Phys. 14, 033001 (2012).

[b24] Steinlechner F., Hermosa N., Pruneri V. & Torres J. P. Frequency conversion of structured light. Sci. Rep. 6, 21390 (2016). 26875448 10.1038/srep21390 PMC4753436

[b25] Boyd R. W. Nonlinear optics (Academic press, 2003).

[b26] Lavery M. P. . Efficient measurement of an optical orbital-angular-momentum spectrum comprising more than 50 states. New J. Phys. 15, 013024 (2013).

[b27] Padgett M. J., Miatto F. M., Lavery M. P., Zeilinger A. & Boyd R. W. Divergence of an orbital-angular-momentum-carrying beam upon propagation. New J. Phys. 17, 023011 (2015).

[b28] Yang Y. . Effect of the radial and azimuthal mode indices of a partially coherent vortex field upon a spatial correlation singularity. New J. Phys. 15, 113053 (2013).

[b29] Yang Y. & Liu Y.-d. Measuring azimuthal and radial mode indices of a partially coherent vortex field. J. Opt. 18, 015604 (2015).

[b30] Hickmann J., Fonseca E., Soares W. & Chávez-Cerda S. Unveiling a truncated optical lattice associated with a triangular aperture using light’s orbital angular momentum. Phys. Rev. Lett. 105, 053904 (2010). 20867921 10.1103/PhysRevLett.105.053904

[b31] Guo C.-S., Lu L.-L. & Wang H.-T. Characterizing topological charge of optical vortices by using an annular aperture. Opt. Lett. 34, 3686–3688 (2009). 19953162 10.1364/OL.34.003686

[b32] Griffiths D. J. & College, R. Introduction to electrodynamics vol. 3 (prentice Hall Upper Saddle River, NJ, 1999).

